# Effect of low-level laser therapy on the membrane induced by the Masquelet technique at an orthotopic site in rabbits

**DOI:** 10.1590/ACB361003

**Published:** 2021-11-22

**Authors:** Jeniffer Gabriela Figueroa Coris, Sheila Canevese Rahal, Carlos Eduardo Fonseca-Alves, Maria Jaqueline Mamprim, Letícia Rocha Inamassu, Alexandre Botelho de Abreu Sampaio, Washington Takashi Kano, Luciane dos Reis Mesquita, José Ivaldo de Siqueira Silva

**Affiliations:** 1 MSc. Postgraduate Program in Animal Biotechnology - Department of Veterinary Surgery and Animal Reproduction - School of Veterinary Medicine and Animal Science – Universidade Estadual Paulista (UNESP) – Botucatu (SP), Brazil.; 2 MSc, PhD. Department of Veterinary Surgery and Animal Reproduction - School of Veterinary Medicine and Animal Science – Universidade Estadual Paulista (UNESP) – Botucatu (SP), Brazil.

**Keywords:** Orthopedics, Infrared Rays, Low-Level Light Therapy, Rabbits

## Abstract

**Purpose:**

To evaluate the low-level laser therapy (LLLT) on the membrane induced by the Masquelet technique in rabbits.

**Methods:**

Twelve Norfolk rabbits at approximately 3 months of age were used. A 1-cm segmental defect was induced in both radii, which were filled with polymethylmethacrylate cylinder. LLLT was used postoperatively in the bone defect of one of the forelimbs every 48 hours for 15 days. Six rabbits were euthanatized on third and sixth postoperative weeks.

**Results:**

In both forelimbs, radiographs showed new bone growth from radius cut ends on the third postoperative week and more advanced stage on the sixth postoperative week. Ultrasound showed induced membrane one week after the surgery. Histologically, there were no significant differences in the semi-quantitative score of inflammation intensity, total number of blood vessels, bone metaplasia, and collagen. The average thicknesses were 2,050.17 and 1,451.96 μm for control membranes and 2,724.26 and 2,081.03 μm for irradiated membranes, respectively, on third and sixth postoperative weeks. Vascular endothelial growth factor A (VEGF-A) and platelet derived growth factor (PDGF) expression were present in the induced membranes of control and irradiated forelimbs, but there was no significant difference.

**Conclusions:**

Based on assessment methods, it was not possible to demonstrate the effect of LLLT on the induced membrane.

## Introduction

The reconstruction of bone defects is one of the greatest challenges in orthopedics, particularly extensive segmental bone defects caused by high-energy accidents or debridement of infected bone fragments^1^^-^ 3. Various reconstruction techniques have been proposed, including bone grafting, bone lengthening, or bone-transport method, among others^2^^,^ 4.

The fresh autogenous cancellous bone graft is considered the gold standard for bone regeneration due to its osteogenic, osteoinductive, and osteoconductive properties^4^. However, to ensure that the graft transferred to a bone defect be capable of performing its functions, studies have been conducted with synthetic polymer membranes^5^, titanium cages^6^, and Masquelet induced membrane technique^7^.

The Masquelet induced membrane technique includes two surgical steps^1^^,^ 3 ^,^ 7 ^,^ 8. The first step comprises the site debridement, followed by the placement of the polymethylmethacrylate (PMMA) – based bone cement spacer in the bone defect^1^^,^ 2 ^,^ 8. The PMMA spacer prevents the interposition of soft tissue in the bone defect^7^, promotes an inflammatory reaction, and induces the development of an encapsulation membrane^2^. In the second step, carried out approximately six to eight weeks later, the cement spacer is removed, but the induced membrane is preserved^7^. Then, the membrane cavity is filled with bone graft, and adjacent soft tissues and membrane are sutured, forming a biological containment system^1^^,^ 7. The induced membrane is capable to prevent bone graft resorption and improves vascularity and corticalization^1^^,^ 9.

Photobiomodulation with low-level laser therapy (LLLT) has been used to improve bone regeneration and bone repair in several conditions, including bone defects and fractures, post-extraction socket, dental implants, among others^10^^-^ 12. After LLLT irradiation, positive effects have been reported, such as increased osteoblastic proliferation, new bone formation, increased deposition of hydroxyapatite and collagen, improvement of the biomechanical properties of bone, and increased expression of genes and bone proteins^10^^,^ 12. Improvement of bone matrix production has been related to anti-inflammatory effects and increased vascularization^10^. In addition, bone reconstruction studies by using guided bone regeneration with different types of membranes have shown increased bone formation after LLLT, with or without biomaterial application for filling the bone defect^13^^,^ 14.

Therefore, this study aimed to evaluate, using radiography, ultrasonography, histology, and immunohistochemistry, the effect of LLLT on the induced membrane (Masquelet technique) at an orthotopic site in rabbits. The hypothesis was that the development of induced membrane could be affected by laser exposure.

## Methods

This study followed the guidelines for the care and use of laboratory animals and was approved by the Institutional Ethics Committee for the Use of Animals (CEUA no. 0209/2018).

### Animals and experimental design

Twelve female Norfolk rabbits, approximately 3 months of age, weighing 2.04–3.13 kg (mean ± standard deviation – SD, 2.57 kg ± 0.35 kg) were used. The rabbits were kept in individual cages and received commercial pelleted diet and water *ad libitum*.

The rabbits were randomly divided according to time points of assessment as follows: six rabbits at three weeks after surgery and six at six weeks after surgery. The rabbits were numbered from 1 to 12. In all rabbits, the right and left segmental bone defects were filled with bone cement. The LLLT was randomly applied to one of the two bone defects.

### Anesthesia and surgical procedure

The rabbits were premedicated with midazolam (2 mg/kg IM) and morphine (2 mg/kg IM) and after 10 minutes received dissociative anesthesia with a combination of ketamine (30 mg/kg IM) and xylazine (0.5 mg/kg IM). General anesthesia was maintained with isoflurane through a facial mask.

**Figure 1:**
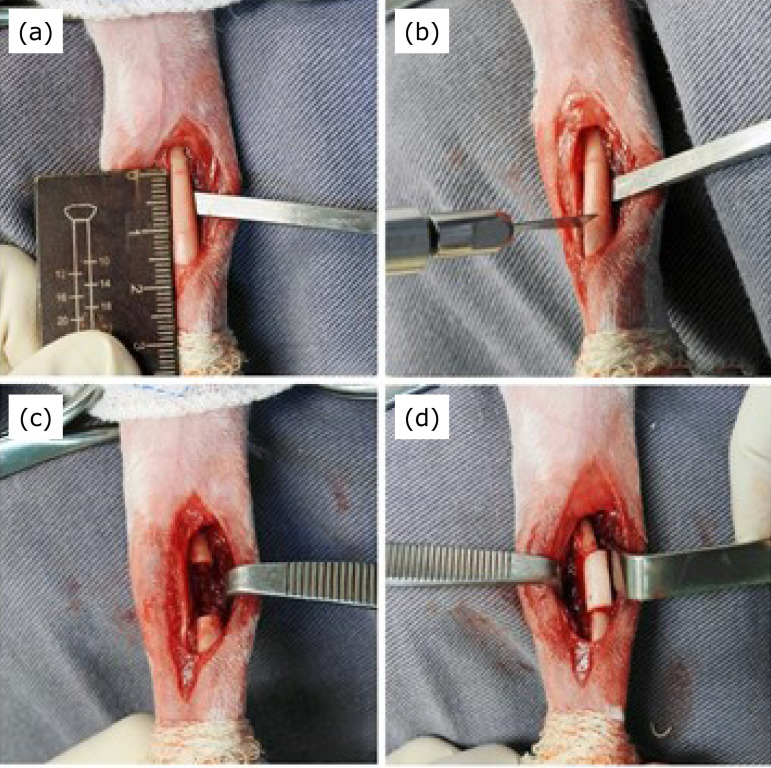
Stage 1 of the Masquelet procedure in a rabbit. **(a)** Radius exposed. **(b)** Bone section using an oscillatory saw. **(c)** 1-cm segmental bone defect. **(d)** Radius defect site filled with a cylinder of polymethylmethacrylate bone cement.

After clipping the hair in both forelimbs, the rabbit was placed in dorsal recumbency. Then, the skin was scrubbed with chlorhexidine acetate solution, and sterile surgical drapes were used to isolate the surgical sites in each forelimb. Using a craniomedial approach, a longitudinal skin incision was made over the proximal third and middle third of the right radius. The extensor carpi radialis and common digital extensor muscles were identified and retracted. The pronator teres muscle was then incised to expose the radius (Fig. 1a). A 1-cm segmental defect, which included the surrounding periosteum, was created in the portion of the proximal to the middle third of radius using an oscillator saw (Fig. 1 b-c). The bone defect was filled with a cylinder of PMMA bone cement (Baumer Ósteo-Class; Mogi Mirim, SP, Brazil), that was made immediately before the surgery (Fig. 1d). The muscles and fascia were closed with 3-0 polyglactin 910 in a simple continuous pattern, as well as the subcutaneous tissue. Simple interrupted suture 3-0 mononylon was used to close the skin. The same procedure was performed on the left radius.

Immediately before the beginning of surgery and in the postoperative period, enrofloxacin (10 mg/kg SC q24h for five days), tramadol chlorhydrate (10 mg/kg SC q24h for five days), and meloxicam (0.5 mg/kg IM q24 hours for three days) were given. The surgical sites were cleaned daily with saline solution. The skin sutures were removed after 10 days postoperative.

### Low-level laser therapy

A low-level gallium-aluminum-arsenide (GaAlAs) diode laser device (Therapy XT, Laser DMC; São Carlos, SP, Brazil) with a continuous wavelength of 808 nm was used. The LLLT device had continuous wave emission, an output power of 100 mW, 2.30 W/cm^2^ power density, beam area of 0.0434 cm^2^, and divergence of 0.45 ± 0.03 rad. The laser application was performed using a punctual technique, with a pen device positioned perpendicularly to the center of the bone defect in one of the forelimbs. Infrared radiation was used for 20 seconds, providing 2 J of energy (46.10 J/cm^2^), every 48 hours, for 15 days. The protocol was calculated by the manufacturer according to the size and depth of the lesion aiming at the induced membrane.

### Clinical, radiographic, and ultrasonographic evaluations

Clinical assessment included weight-bearing on the operated forelimbs and wound healing.

Craniocaudal and mediolateral projections of the right and left antebrachium were taken immediately after surgery and on the third and sixth postoperative weeks. A digital radiography system (DR-F, GE Healthcare Systems; Barueri, SP, Brazil) was used to obtain the images, with 1-m focal*-*film distance and settings of 45 kV, 8 mAs, and 200 mA. The position of the PMMA cylinder in the bone defect, tissue reaction, and signs of bone proliferation (present or absence) were evaluated.

B-mode and color Doppler ultrasound imaging were performed immediately after surgery and every seven days, to identify the presence of induced membrane, as well as the presence or absence of local neovascularization. Ultrasound equipment equipped with an 8-13 MHz linear transducer (LOGIQ-e; GE Healthcare Systems; Barueri, SP, Brazil) was used. The images were obtained in sagittal and medial planes.

### Macroscopic, histological, and immunohistochemical analyses

Six rabbits were euthanized at the third week and at the sixth week after surgery to proceed with the histological analysis. The rabbits received ketamine (50 mg/kg) and xylazine (1 mg/kg) intramuscularly, followed by 19.1% potassium chloride intravenous overdose until cardiorespiratory arrest.

The induced membranes were carefully harvested and macroscopically evaluated, fixed in 10% buffered formalin for 48 hours, and followed by storage in 70% alcohol. After dehydration, clarification, and impregnation steps, the induced membranes were embedded in paraffin. Then, 5-μm sections were cut from the paraffin blocks and stained with hematoxylin and eosin (H&E). The induced membranes were assessed for cellular characteristics and the presence of angiogenesis. A semi-quantitative scoring was used to assess the amount of inflammatory cells^15^:

- Grade 0: absence of inflammatory cells;
- Grade 1 (mild): less than 25%;
- Grade 2 (moderate): from 26 up to 50%;
- Grade 3 (intense): more than 51%.

The vessels were counted using a 10 × 10 grid reticle at x40 magnification.

The measurement of the thickness of induced membranes was taken on a microscope (Olympus BX 53) equipped with a camera (DP-26) and an image analysis system (cellSens Standard). All measurements were performed in triplicate in three regions (left, central, and right) using a x10 ocular lens.

For immunohistochemistry evaluation, the paraffin-embedded tissue blocks were sectioned into 3-μm-thick sections and mounted on coated glass slides (StarFrost, Knittel; Braunschweig, Germany). The slides were dewaxed with xylol and rehydrated in decreasing concentrations of ethanol to distilled water. Endogenous peroxidase blockade was performed with 8% hydrogen peroxide diluted in methyl alcohol. Antigen recovery was performed with citrate buffer (pH = 6) using a pressure cooker (Pascal, Dako; Carpinteria, CA, United States of America) for 10 minutes.

Mouse vascular endothelial growth factor A (VEGF-A) monoclonal antibody at a dilution of 1:300 (Flk1, Abcam; Cambridge, United Kingdom) and rabbit monoclonal Platelet-derived growth factor (PDGF) (clone 26E1, Cell signaling; Danvers, MA, United States of America) at a dilution of 1:200 were used for 18 hours.

Subsequently, the sections were incubated with secondary antibody (Envision, Dako; Carpinteria, CA, United States of America) for 1 hour, and 3.30-diaminobenzidine (DAB, Dako; Carpinteria, CA, United States of America) for 5 minutes. Counterstaining with Harris’ Hematoxylin was done for 1 minute. Positive controls were determined according to Human Protein Atlas guidelines (https://www.proteinatlas.org). The canine liver was used as a positive control for VEGF-A, and the canine testicle was used as a positive control for PDGF. The samples were evaluated by light microscopy with a semi-quantitative score as follows^15^:

- 0: absence of reactivity;
- 1: 1 to 25% of positive cells;
- 2: 26 to 50% of positive cells;
- 3: 51 to 75% of positive cells;
- 4: > 75% positive cells for distribution.

Intensity score was determined as: absence (0), mild (1), moderate (2), intense (3).

### Statistical analysis

Continuous variables were assessed using normality tests (Shapiro-Wilk) and graphical analysis (histogram and QQ plot). Chi-square and Fisher tests were used to verify the association between type of treatment (LLLT or control) and occurrence of different categorical variables (presence of vessels on ultrasound examination, presence of bone metaplasia, and collagen on histological examination) for each time point. For the histological variables (inflammatory score, number of vessels) and immunohistochemistry variables (distribution and intensity of VEGF-A and PDGF), the paired Wilcoxon test was used to compare the median of scores for the irradiated membranes and control membranes at each time point, and the Kruskal-Wallis test was used to compare the median at each time point within each treatment.

The average thickness of the membrane was compared between the different time points and treatments using an analysis of variance followed by Tukey. The statistical analyses were accomplished with GraphPad Prism (GraphPad Software Inc.; La Jolla, CA, United States of America) and Statistical Analytical software (SAS Institute, 2011). Differences were considered statistically significant at P *<* 0.05.

## Results

### Clinical, radiographic, and ultrasonographic evaluations

The wounds healed despite the partial dehiscence of the wound in two forelimbs. The weight-bearing on the forelimbs occurred approximately 12 hours after surgery. All rabbits showed normal eating and drinking during the study.

**Figure 2:**
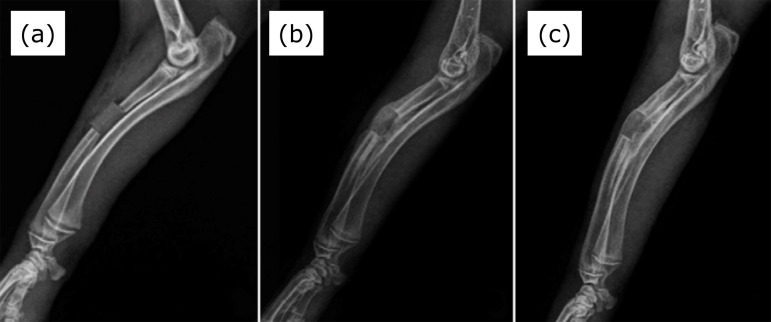
Mediolateral radiographic views of the rabbit forelimbs that received low*-*level laser therapy in the immediate postoperative **(a)**, and at third **(b)** and sixth weeks after induced Masquelet membrane. Observe the segmental bone defect filled with a polymethylmethacrylate cylinder **(a)**, new bone growth from the cut ends of the radius **(b)**, and remodeling of the bony callus **(c)**.

In both forelimbs, radiographs showed new bone growth from the cut ends of the radius on the third postoperative week (Fig. 2). On the sixth postoperative week, bone growth from the cut ends was more intense in three control forelimbs (50%) and two forelimbs irradiated with LLLT (33.3%). One control forelimb (17.7%) and one irradiated forelimb (17.7%) showed bridging of bone over the defect. Remodeling of the bony callus was seen in two control forelimbs (33.3%) and three irradiated forelimbs (50%) (Fig. 1). Including both time points, the cylinders showed a slight degree of displacement on postoperative radiographs in 75% of the control forelimbs and 83.3% in irradiated forelimbs.

**Figure 3:**
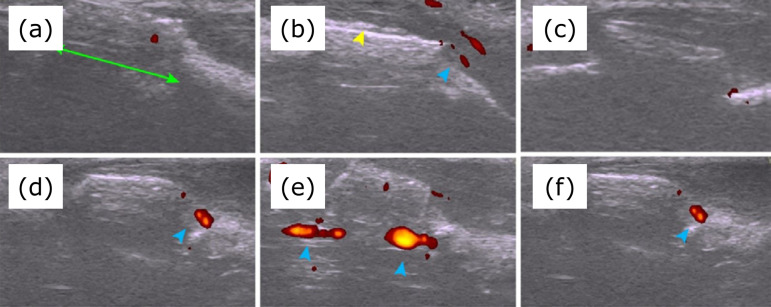
Ultrasound images in the longitudinal plane of a segmental defect in the radius of rabbits filled with a polymethylmethacrylate cylinder, with 1 **(a)**, 2 **(b)**, 3 **(c)**, 4 **(d)**, 5 **(e)**, and 6 **(f)** postoperative weeks. In B-mode ultrasonography, a rectilinear structure with a hyperechogenic surface forming posterior acoustic shading is observed, corresponding to the polymethylmethacrylate cylinder (*green arrow*) and the formation of the induced membrane (*yellow arrow*). In Power-Doppler mode, the presence of blood vessels is visible near the bone surface and cement, representing vascularization of the induced membrane (*blue arrow*).

Ultrasound assessment of the bone defect with Doppler identified the PMMA spacer in all 24 forelimbs in the immediate postoperative period. One week after the surgery, the presence of the induced membrane was seen in both control and irradiated forelimbs. The presence of vascularization in the region of the induced membrane according to the forelimb and time points are described in Table 1 and illustrated in Fig. 3. No statistically significant difference was found between control and irradiated forelimbs.

**Table 1:** Occurrence (and percentage) of vessels evaluated by color Doppler ultrasound on induced membrane immediately after surgery (0) and from one to the sixth week of postoperative, according to the number of forelimbs (6 or 12).

Time points	Control	Irradiated with low-level laser therapy
0	0/12 (0%)	1/12 (8.33%)
1	7/12 (58.33%)	6/12 (50%)
2	7/12 (58.33%)	7/12 (58.33%)
3	8/12 (66.67%)	6/12 (50%)
4	3/6 (50%)	4/6 (66.67%)
5	3/6 (50%)	3/6 (50%)
6	2/6 (33.33%)	3/6 (50%)

### Macroscopic, histological, and immunohistochemical analyses

In the macroscopic evaluation on the third week after surgery, the induced membranes were easily identified and collected, since there was no adherence to the PMMA cylinder. The induce membrane had a rough appearance in its outermost and smoothest portion in direct contact with the cement. In the forelimbs that received LLLT, the membrane was thicker than the control. On the sixth postoperative week, the control and irradiated membranes were thin and adhered to adjacent tissues, which made dissection difficult.

**Figure 4:**
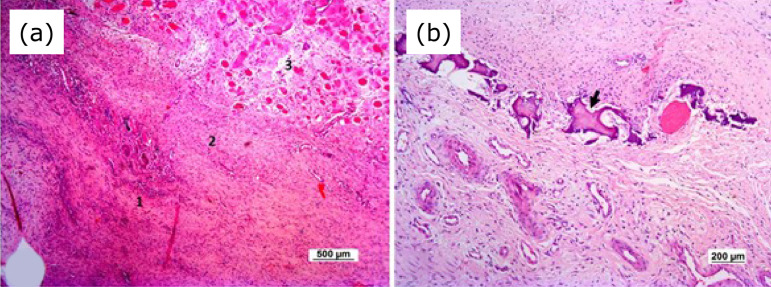
Photomicrographs of the induced membrane that received low*-*level laser therapy on the third postoperative week **(a)** and on the sixth postoperative week **(b)**. **(a)** Note first layer (1) in contact to PMMA cylinder, composed of mononuclear and polymorphonuclear inflammatory cells and blood vessels; second layer (2) with linear fibroblasts oriented parallel to the PMMA cylinder, presence of organized collagen fibers, and blood vessels; third layer (3) constituted by disorganized connective tissue, active fibroblasts, and blood vessels (HE, x50). **(b)** Observe just one layer with a presence of bone trabeculae (black arrow) (H&E, x200).

Two induced membranes of one rabbit on the sixth postoperative week were excluded from the analysis because of a processing problem in histology. Three distinct layers were observed in four control membranes (66.7%) and three irradiated membranes (50%) on the third postoperative week, as well as in one control membrane (20%) and one irradiated membrane (20%) on the sixth postoperative week (Fig. 4a). The first layer in contact with the PMMA cylinder had mononuclear and polymorphonuclear inflammatory cells and blood vessels. The second layer showed an abundant amount of linear fibroblasts oriented parallel to the PMMA cylinder, the presence of organized collagen fibers, and blood vessels. The third layer in contact with adjacent musculature consisted of disorganized connective tissue, active fibroblasts, and blood vessels. Two distinct layers were observed in two control membranes (33.33%) and one irradiated membrane on the third postoperative week (16.67%), as well as two control membranes (40%) and one irradiated membrane (20%) on the sixth postoperative week. Just one layer was verified in two irradiated membranes (33.33%) on the third postoperative week, likewise two control (40%) and three irradiated (60%) membranes on the sixth postoperative week that had bone trabeculae (Fig. 4b).

PMMA: polymethylmethacrylate; H&E: hematoxylin and eosin.

The histological analysis of the central portion of the induced membranes on the third postoperative week showed that all control membranes (100%) had vascularization, and irradiated membranes had the presence of vascularization in five of them (83.3%), and absence of vascularization in one (16.7%), that was composed of bone trabeculae. The inflammatory score ranged from 1 (83.3%) to 2 (16.7%) in the control membranes and from 0 (50%) to 1 (50%) in the irradiated membranes. Bone metaplasia was detected in all (100%) control membranes and five irradiated membranes (83.3%). Collagen was found in five (83.3%) control membranes and three (50%) irradiated membranes. The histological analysis on the sixth postoperative week showed that all control membranes (100%) had vascularization, and irradiated membranes had the presence of vascularization in four of them (80%). The inflammatory score ranged from 0 (60%) to 1 (40%) in both control and irradiated membranes. Bone metaplasia was detected in two (40%) control membranes and three irradiated membranes (60%). Collagen was found in three (60%) control membranes and two (40%) irradiated membranes. There were no statistically significant differences in the variables between control and irradiated membranes on third and sixth postoperative weeks (Table 2).

**Table 2:** Semi-quantitative inflammatory score, total number of blood vessels, presence or absence of bone metaplasia and collagen on histological evaluation of induced membrane, irradiated or not with low-intensity laser, in six rabbits on the third postoperative week and in five rabbits on the sixth postoperative week.

Variables	Membranes	Third postoperative week									P value	Sixth postoperative week								
		A1	A2	A3	A4	A5	A6	Min	Max	Median		A7	A8	A9	A10	A11	Min	Max	Median	P value
Inflammatory score *	control	1	1	2	1	1	1	1	2	1	0.1250	1	0	1	0	0	0	1	1	0.9999
	laser	0	1	1	1	0	0	0	1	0.5		1	0	1	0	0	0	1	0	
Blood vessels	control	7	15	16	5	11	11	5	16	11	0.1875	9	11	4	7	3	3	11	7	0.6250
	laser	3	14	10	11	2	0	0	14	6.5		4	9	7	1	7	1	9	7	
Bone metaplasia	control	1	1	1	1	1	1	1	1	-	1.0000	0	1	1	0	0	1	1	-	1.0000
	laser	1	1	1	1	1	0	0	1	-		1	0	1	0	1	0	1	-	
Collagen	control	1	1	1	1	1	0	0	1	-	0.0606	1	0	0	1	1	0	1	-	1.0000
	laser	1	0	1	0	0	1	0	1	-		0	0	1	1	0	0	1	-	

* Inflammatory score: absence (grade 0), mild (grade 1), moderate (grade 2), intense (grade 3). Bone metaplasia: absence (0), presence (1). Collagen: absence (0), presence (1); A: animal; Min: minimum; Max: Maximum.

The average thicknesses of the induced membranes, including the three areas of measurement, were 2,050.17 μm (± 600.28 μm) for control membranes and 2,724.26 μm (± 737.46 μm) for irradiated membranes on the third postoperative week, and 1,451.96 μm (± 265.07 μm) for control membranes and 2,081.03 μm (± 1,227.61) for irradiated membranes on the sixth postoperative week. There was no statistically significant difference between treatments.

**Figure 5:**
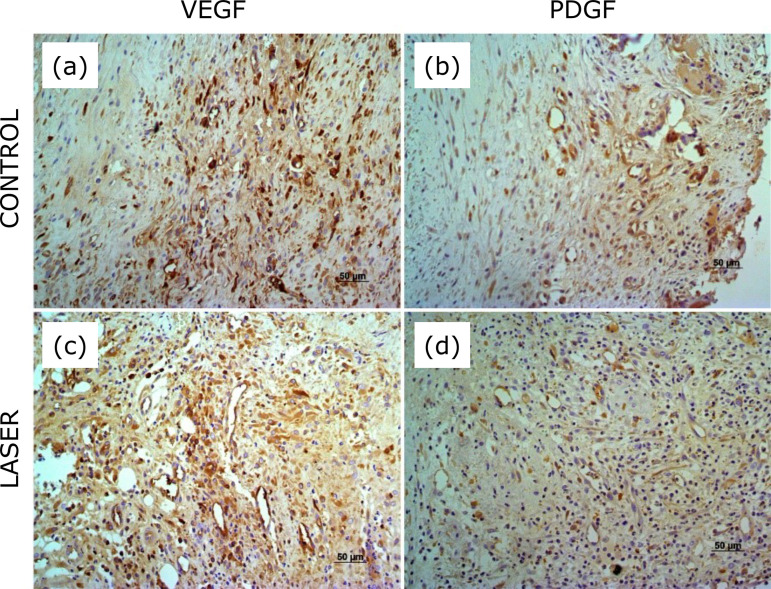
Immunohistochemical staining for VEGF-A and PDGF in the induced membrane on the third postoperative week. **(a)** Control membrane shows distribution 3 score and intensity 1 score. **(b)** The membrane that received low*-*level laser therapy shows distribution 2 score and intensity 1 score. **(c)** Control membrane shows distribution 4 score and intensity 2 score. **(d)** The membrane that received low*-*level laser therapy shows distribution 3 score and intensity 2 score.

VEGF: Vascular endothelial growth; PDGF: platelet derived growth factor; VEGF-A: Vascular endothelial growth factor A.

**Figure 6:**
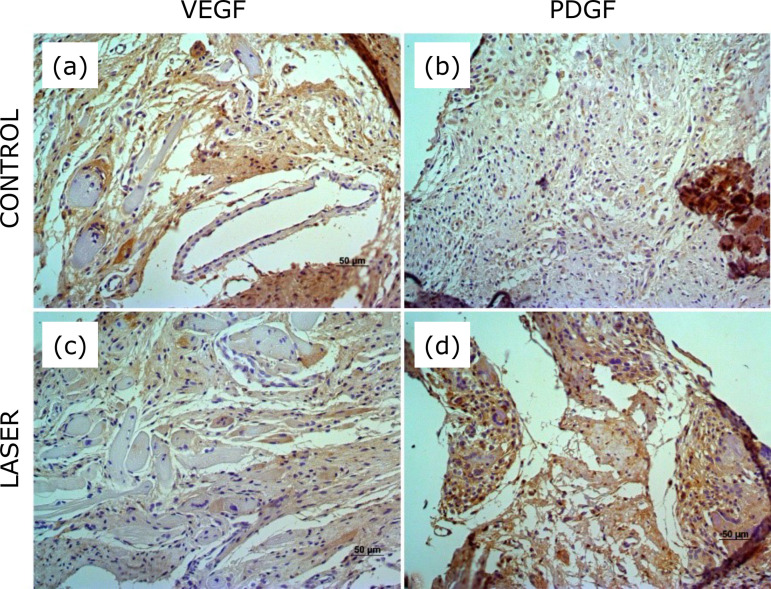
Immunohistochemical staining for VEGF-A and PDGF in the induced membrane on the third postoperative week. **(a)** Control membrane shows distribution 2 score and intensity 1 score. **(b)** The membrane that received low*-*level laser therapy shows distribution 3 score and intensity 2 score. **(c)** Control membrane shows distribution 2 score and intensity 1 score. **(d)** The membrane that received low*-*level laser therapy shows distribution 3 score and intensity 2 score.

VEGF: Vascular endothelial growth; PDGF: platelet derived growth factor; VEGF-A: Vascular endothelial growth factor A.

VEGF-A expression was present in control and irradiated membranes (Figs. 5 and 6). On the third week postoperative, the distribution score varied from 2 (66.7%) to 3 (33.3%) and intensity score from 1 (66.7%) to 2 (33.3 %) in control membranes. In irradiated membranes, the distribution score varied from 1 to 3, one (16.7%) with score 1, two (33.3%) with score 2, and three (50%) with score 3, and the intensity varied from 1 (33.3%) to 2 (66.7%). On the sixth week postoperative, VEGF-A expression showed a distribution of score 2 and intensity of score 1 in all control membranes, and distribution from 1 (40%) to 2 (60%) and intensity of score 1 (100%) in all irradiated membranes. There was no statistically significant difference between control and irradiated membranes (Table 3).

PDGF expression was present in control and irradiated membranes (Figs. 5 and 6). On the third week postoperative, the distribution score varied from 2 (50%) to 3 (50%) and the intensity score from 1 to 2, two (33.3%) with score 1 and four with score 2 (66.7%) in control membrane, and three (50%) with score 1 and three (50%) with score 2 in the irradiated membrane. On the sixth week postoperative, PDGF expression showed a distribution score from 2 (40%) and 3 (60%) and intensity score from 1 (40%) and 2 (60%), in both control and irradiated membranes. There was no statistically significant difference between control and irradiated membranes (Table 3).

**Table 3:** Semi-quantitative scores of the immunohistochemical staining for vascular endothelial growth factor (VEGF) and platelet-derived growth factor (PDGF) in induced membranes, irradiated or not with low-intensity laser, in six rabbits on the third postoperative week and in five rabbits on the sixth postoperative week.

Variables	Membranes	Third postoperative week										Sixth postoperative week								
		A1	A2	A3	A4	A5	A6	Min	Max	Median	P value	A7	A8	A9	A10	A11	Min	Max	Median	P value
VEGF distribution	Control	2	3	3	2	2	2	2	3	2.5	0.9999	2	2	2	2	2	2	2	2	0.5000
	Laser	1	2	2	3	2	3	1	3	2		1	1	2	2	2	1	2	2	
VEGF intensity	Control	1	1	2	2	1	1	1	2	1	0.6250	1	1	1	1	1	1	1	1	1.0000
	Laser	1	2	1	2	2	2	1	2	2		1	1	1	1	1	1	1	1	
PDGF distribution	Control	3	3	3	2	2	2	2	3	2.5	0.9999	3	3	3	2	2	2	3	3	0.9999
	Laser	2	2	3	2	3	3	2	3	2.5		2	3	3	3	2	2	3	3	
PDGF intensity	Control	2	2	2	2	1	2	1	2	2	0.9999	2	1	2	1	2	1	2	2	0.9999
	Laser	1	1	2	2	1	2	1	2	1.5		2	2	2	1	1	1	2	2	

## Discussion

This study evaluated the influence of LLLT on the induced membrane, and there were no significant differences in histological characteristics compared to the control.

Although the periosteum was resected, the 1-cm bone defect could not be considered critical due size defect and age of the rabbits^16^. Despite this, membrane development could be evaluated. The bone cement was placed as a pre-molded cylinder in diameter larger than the segmental bone defect to obtain a large size induced membrane, supposing that it would facilitate the second step of the technique, which consists of cement spacer removed and filling inside the membrane with cancellous bone graft, bone substitutes, or combinations^1^^,^ 8 ^,^ 9.

In human patients, the shape of the spacer has been applied as cylinders, beads, and flat pebbles, but the most common is as cylinder applied before its solidification^8^. The cylinder showed a slight degree of displacement on postoperative radiographs of the forelimbs, which did not interfere with the membrane development, based on ultrasound findings, as well as macroscopic and histological analyses. The wrapping of the PMMA in the dough stage around the bone ends, and cylinder probably would prevent cylinder displacement. No interference was observed in the second step of the induced membrane, in a rabbit study that used PMMA around bone ends and to fill the ulna defect^17^.

In addition, in both control and irradiated bone defect new bone growth was seen from the cut ends of the radius on the third postoperative week, compatible to fracture stabilized by a non-rigid fixation, typically characterized by secondary bone healing^18^. In its turn, on the sixth postoperative week, evolution of the process was found, but no statistical difference between control and irradiated forelimbs was verified.

Since the rabbits were young, the induced membrane was well-developed on the third postoperative week according to macroscopic and histological examinations, in both control and irradiated forelimb. In human patients, a time of around six to eight weeks has been recommended for the development of the induced membrane^1^^,^ 4 ^,^ 9. In studies with rabbits, the formation time of induction membrane has been reported from four to six weeks, but the differences in age, size of bone defect, and type of immobilization must be considered^17^^,^ 19 ^,^ 20.

Three distinct layers were observed in seven induced membranes on the third postoperative week and two on the sixth postoperative week, being five control and four irradiated membranes. In rats three distinct layers were also identified from three to six weeks after PMMA insertion^21^. However, this distinction was not evident in the other induced membranes in the present study, which had two or one layers. In addition, some irradiated membranes had the presence of bone trabeculae, suggesting a more advanced stage of cell differentiation. Laser therapy is characterized by helping in the tissue repair process by altering cellular behavior and increasing vascular formation, along with the production of collagen and fibroblasts^11^.

The vascularization of the induced membranes, as well as histological evaluations, was detected by Doppler ultrasound. However, vascularization was lower in irradiated membranes compared to control membranes. Laser therapy, especially with infrared wavelengths, appears to improve bone matrix production by increasing vascularization and anti-inflammatory effects. However, the effect is more effective if the treatment is carried out during the initial phase when high cell proliferation occurs^10^. The lower vascularization in irradiated membranes may suggest that these induced membranes were into a more advanced stage. In a rabbit study, a gradual reduction in vascular density of induced membrane was also observed^22^. Thus, the second stage of the technique could be carried out before the sixth week of membrane induction. A highly vascularized membrane in all layers is essential for the success of the second stage since the membrane stimulates vascularization and corticalization of cancellous bone^1^.

The immunohistochemistry detected, in both control and irradiated membranes, the presence of VEGF-A, that is responsible for local angiogenesis and endothelial cell proliferation and migration^23^, as well as PDGF, that induces cell proliferation, chemotaxis and extracellular matrix synthesis, contributing to tissue regeneration^24^^,^ 25. Other studies in rabbits have also shown the ability of induced membranes to secrete growth factors^22^^,^ 26. The growth factors were detected in the induced membranes at the third and the sixth week. In a study with rabbits, high concentrations of VEGF and TGF-ß1 were found at the second week of membrane induction and remained stable at the fourth, sixth, and eighth postoperative weeks^26^. However, in a study with human patients, VEGF, IL-6 and Col-1 had high expression in the induced membrane at the end of one month, but the levels were below 40% in induced membranes at the second month^27^.

The levels of growth factors did not show a significant difference in the comparison between control and irradiated membranes. However, some studies have shown that laser therapy is able to promote the increase or biomodulation of growth factors, such as VEGF, FGF-2, EGF, PDGF, and TNF-α, and TGF-ß^28^^-^ 30. However, in rat calvarial defects filled with xenograft and covered with the collagen membrane, the photobiomodulation provided a greater amount of bone volume only during the first 14 days after surgery^14^. Thus, assessments at earlier time points than those used in the present study would be important to determine the validity of this premise.

Even though there were no statistically significant differences, histologically the irradiated membranes had an average of 2,724.26 and 2,081.03 μm at the third and at the sixth week in comparison to 2,050.17 and 1,451.96 μm of the control membrane at the same time points. The laser irradiation showed some degree of action on tissue proliferation. A study mentioned that laser therapy helps in the tissue repair process because of cellular behavior alteration and increasing of vascular formation, production of collagen, fibroblasts, and epithelial tissue^11^.

The induced membrane was less thick at the sixth postoperative week, both in control and irradiated membranes, suggesting that this time point would be inappropriate for the second stage of the procedure. Likewise, a study in rabbits also observed a decrease in the induced membrane thickness, with averages of 1,360, 1,045, 1,015 and 1,008 μm, corresponding to the second, fourth, sixth and eighth postoperative week, respectively^22^.

It must also be considered that the effect of photobiomodulation on bone regeneration depends on the irradiation dose, as well as on the duration and mode of irradiation^10^. Since the present study used only one irradiation protocol, this may be considered a limitation. Thus, further studies using different irradiation doses need to be carried out.

## Conclusion

Based on imaging studies and histology and immunohistochemistry, the LLLT at 2 J of energy for 20 seconds, every 48 hours for 15 days, did not show effects on the development of induced membrane at an orthotopic site in rabbits compared to non-irradiated control ones.
